# miR-376a Provokes Rectum Adenocarcinoma *Via* CTC1 Depletion-Induced Telomere Dysfunction

**DOI:** 10.3389/fcell.2021.649328

**Published:** 2021-04-16

**Authors:** Yang Liu, Xiaotong Zhao, Bing Wang, Zhijia Liu, Manman Zhang, Jinhan Wang, Chang Xu, Yan Wang, Liqing Du, Feng Wang, Qin Wang, Qiang Liu

**Affiliations:** ^1^Tianjin Key Laboratory of Radiation Medicine and Molecular Nuclear Medicine, Institute of Radiation Medicine, Chinese Academy of Medical Sciences, Peking Union Medical College, Tianjin, China; ^2^Department of Genetics, School of Basic Medical Sciences, Tianjin Medical University, Tianjin, China

**Keywords:** CST, telomere, rectum adenocarcinoma, miR-376a, microRNA

## Abstract

CTC1 is a component of the mammalian CST (CTC1–STN1–TEN1) complex which plays essential roles in resolving replication problems to facilitate telomeric DNA and genomic DNA replication. We previously reported that the depletion of CTC1 leads to stalled replication fork restart defects. Moreover, the mutation in CTC1 caused cancer-prone diseases including Coats plus (CP) or dyskeratosis congenita (DC). To better understand the CTC1 regulatory axis, the microRNAs (miRNAs) targeting to CTC1 were predicted by a bioinformatics tool, and the selected candidates were further confirmed by a dual-luciferase reporter assay. Here, our current results revealed that miR-376a significantly reduced CTC1 expression at the transcription level by recognizing CTC1 3′-UTR. In addition, the overexpression of miR-376a induced telomere replication defection and resulted in direct replicative telomere damage, which could be rescued by adding back CTC1. Telomere shortening was also observed upon miR-376a treatment. Furthermore, for the clinical patient samples, the high expression of miR-376a was associated with the deregulation of CTC1 and a poor outcome for the rectum adenocarcinoma patients. Together, our results uncovered a novel role of miR-376a in stimulating rectum adenocarcinoma progression *via* CTC1 downregulating induced telomere dysfunction.

## Introduction

Telomere is a tandemly repeated DNA formed at the end of a chromosome and is thought to play essential roles in genomic stability (Turner et al., 2019). Given the end replication problems, telomeres shorten with cell division, and the cells will go into senescence or apoptosis when their telomere reaches critical lengths that are no longer enough to cap the essential genes on the genome (Zhu et al., 2019). Telomere length could be maintained by the telomerase *via* adding TTAGGG repeats to the chromosome ends, and the activity of telomerase, which is a ribonucleoprotein reverse transcriptase, could be detected in a variety of cells, such as cancer cells, stem cells, and reproductive cells (Jiang et al., 2018).

Telomere proteins protect chromosome termini to distinguish the natural ends from double-stranded breaks to further inhibit processes such as DNA end-joining, DNA recombination, or DNA repair, which would lead to unstable chromosomes (Yin et al., 2014). Shelterin is the conserved telomere binding complex consisting of six distinct proteins, including TRF1, TRF2, Rap1, TIN2, TPP1, and POT1 (Yin et al., 2014). TRF1 and TRF2 bind to the duplex telomere with high affinity, playing essential roles in telomere replication and protection. POT1 is the single-version component of shelterin that binds to the single-strand telomeric DNA and functions in telomere protection, G-overhang processing, and telomerase activity regulation. Rap1, TIN2, and TPP1 connect and stabilize the above three proteins on telomeres (Yin et al., 2014; Liu et al., 2019).

Telomeres are difficult-to-replicate sites due to their repetitive nature and their propensity to generate DNA secondary structure, such as T-loop and G-quadruplex. Moreover, the heterochromatic states provide an additional barrier to the replication forks (Bettin et al., 2019). Thus, a number of extra protein factors are required to properly replicate the telomeric duplex and to pass through the inherent barrier. These proteins include DNA helicase (BLM, RTEL1, and RecQ4) (Root et al., 2016; Kaiser et al., 2017; Olivier et al., 2018), nuclease (FEN1 and Dna2) (Saharia et al., 2010; Lin et al., 2013), and DNA binding proteins (hSSB) (Gu et al., 2013; Touma et al., 2016). The depletion of these proteins leads to telomere dysfunction, represented by the formation of multi-telomeric signals (MTSs) and telomere dysfunction-induced foci (TIFs). Our previous studies showed that mammalian CST (CTC1–STN1–TEN1) plays essential roles in stalled telomeric replication fork restart and the telomeric C-strand fill-in process (Zhang M. et al., 2019). The depletion of CTC1 caused the appearance of telomere replication deficiency and the accumulation of telomere damage (Feng et al., 2018). Moreover, a naturally occurring mutation of CTC1 was observed in rare genetic telomere biology disorders (TBD) such as Coats plus (CP) or dyskeratosis congenita (DC), of which patients always show shortened telomeres (Gu et al., 2018; Han et al., 2020).

Recently, a variety of studies have indicated that microRNAs (miRNAs) could play essential roles in a diversity of biological processes (Liu and Liu, 2013; Vinchure et al., 2020). To better understand the role of miRNAs in telomere function, further investigation on the regulatory pathway of CTC1 *via* miRNAs was explored in our present study. Firstly, the miRNAs targeting to CTC1 were screened by using the bioinformatics prediction tool ENCORI. Then, the candidates were identified and confirmed by a dual-luciferase reporter system. Two of the 11 tested miRNAs were found to function in telomere replication and telomere length regulation by downregulating CTC1. Moreover, we also observed that the decrease of CTC1 was consistent with the stimulation of miR-376a in the progression of rectum adenocarcinoma. Together, these findings revealed a novel function of miR-376a and uncovered a universal mechanism of rectum adenocarcinoma generation which relies on telomere length regulation, suggesting a novel potential target for cancer therapy.

## Results

### Bioinformatics Analysis and Experimental Screening Identifies miRNAs Targeting to CTC1

**FIGURE 1:**
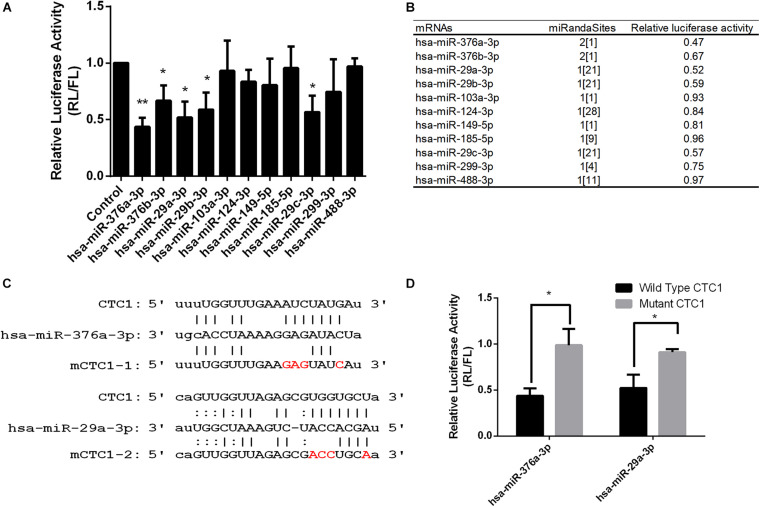
Several microRNAs (miRNAs) were identified to target CTC1. **(A)** Dual-luciferase assay report of HEK293T cells that expressed the indicated miRNA targeting the CTC1 3′-UTR. **(B)** List of candidate miRNAs tested by the luciferase assay and the number of miRanda screening read sites. **(C)** Predicated binding sites of miR-376a and miR-29a on the CTC1 3′-UTR. Mutant sites in the seed region were labeled in *red*. **(D)** Luciferase activity of cells expressing the wild-type CTC1 or mutant CTC1 3′-UTR with the selected miRNAs. *P* values were determined by Student’s *t* test. An asterisk was annotated on the panel if the statistical *P* < 0.05 compared to the control.

To investigate and obtain the candidate miRNAs targeting to CTC1, the bioinformatics prediction tool ENCORI (the Encyclopedia of RNA Interactomes) was used for the first round of screening. ENCORI is an open-source platform for studying the miRNA–target interactions from CLIP-seq, degradome-seq, and RNA–RNA interactome data (Yang et al., 2011; Li et al., 2014). One hundred seven miRNAs were identified to interact with CTC1 messenger RNAs (mRNAs), and these candidates were then re-selected by the miRanda program (Betel et al., 2010). Finally, 11 miRNAs with more miRanda sites read numbers were chosen for the following study (Figure 1A). Given that all of these 11 miRNAs were targeted to the CTC1 3′-UTR, the UTR region of CTC1 (NM_025099.6) was cloned into downstream of the Renilla luciferase (RL) reporter and a dual-luciferase reporter assay was performed to investigate the regulation function of the candidate miRNA. The luciferase vector and the miRNAs were co-transfected into human embryonic kidney 293 (HEK293) cells. The RL/firefly luciferase (FL) rate was used to determine the effect for the corresponding miRNA. The results showed that five of the 11 miRNAs decreased the RL/FL rate, suggesting that they may suppress CTC1 expression through interaction with its 3′-UTR regions (Figure 1B). Among these five miRNAs, miR-376a-3p and miR-29a-3p exhibited better repression efficiency. To further confirm their regulation function, the putative interaction sites of each miRNA were predicted. Two 3′-UTR mutations (mCTC1-1 and mCTC1-2), which can disrupt the miRNA–mRNA interaction, were cloned by quick-change PCR (Figure 1C). As we expected, the overexpression of miR-376a-3p or miR-29a-3p in HCT116 cells could decrease Renilla luciferase activity with the CTC1 3′-UTR wild-type vector, but not for the mutant vectors, confirming that both miRNAs target the 3′-UTR regions (Figure 1D).

### miRNAs Induce Telomere Replication Dysfunction by Downregulating Endogenous CTC1 Protein

**FIGURE 2:**
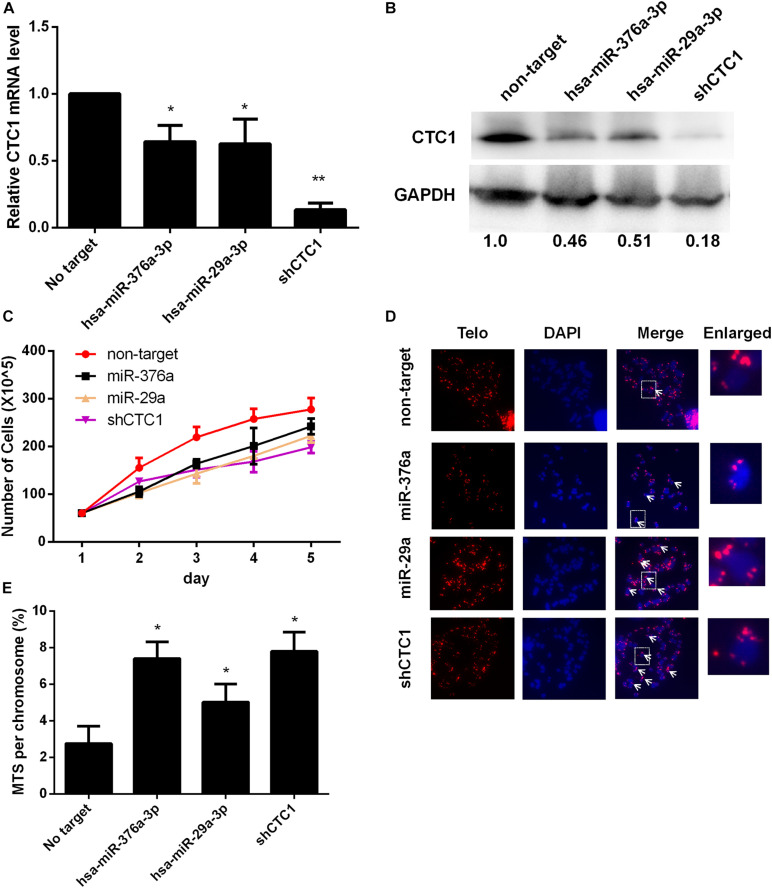
Selected microRNAs (miRNAs) could induce telomere dysfunction *via* downregulating CTC1 expression. **(A)** The relative CTC1 messenger RNA (mRNA) transcriptional level was determined by quantitative real-time PCR in the control and the miRNA or short hairpin RNA (shRNA) treatment groups. The expression difference was calculated by one-way ANOVA, with *P < 0.05 and **P < 0.01. **(B)** The protein level was evaluated by Western blot assay upon miRNA or shRNA treatment. The relative gray value was annotated *below each panel*. **(C)** The growth curve upon miRNA or shRNA treatment was determined by cell counting. **(D)** Representative images of metaphase telomere fluorescence *in situ* hybridization (FISH) upon different treatments. Multi-telomeric signal (MTS) is indicated by the *white arrows*. The *red florescence* exhibits telomere FISH signal while chromosomes were stained with DAPI (*blue*). **(E)** The percentage of chromosomes with MTS was calculated upon miRNA or shRNA treatment based on the FISH results as demonstrated in panel **(D)**. Statistical analysis was calculated by one-way ANOVA, with *P < 0.05.

To investigate whether the expressions of the above miRNAs could inhibit the expression of endogenous CTC1, the level of CTC1 was determined at the mRNA and protein levels upon exogenous miRNA transfection. Our results showed that the mRNA transcription level was reduced by 35.7 and 37.3% with miR-376a-3p and miR-29a-3p treatments, respectively (Figure 2A). Consistently, the CTC1 protein level was decreased by ∼50% by these two miRNAs (Figure 2B), indicating that the expression of the miRNA could downregulate the level of CTC1. Here, the shCTC1 vector was used as a positive control, which showed slightly better inhibition efficiency. Since CTC1 has been reported to play roles in cell growth and cell cycle (Feng et al., 2018), cell proliferation was examined by cell counting. We found that treatment with either of the two miRNAs or by shCTC1 knockdown could finally lead to the repression of cell growth (Figure 2C).

Given the crucial role of CTC1 in telomere replication (Stewart et al., 2012), the level of MTSs (multiple telomere signals) was determined consequently. MTSs have also been called fragile telomeres, which are observed under conditions of replication fork stalling and replication stress, thereby constituting a sign of telomere replication failure (Durkin and Glover, 2007). When we examined the HEK293T stable miRNA expression cell lines, we found that either miR-376a-3p or miR-29a-3p overexpression caused a significant increase in the frequency of MTSs (Figures 2D,E). The acute knockdown of CTC1 led to an increase in chromosomes lacking telomeric fluorescence *in situ* hybridization (FISH) signals (signal-free ends, SFEs) (Surovtseva et al., 2009). We next examined whether stable miRNA expression causes loss of telomere signal. In contrast to the MTS result, no significant increase of SFEs was observed (Supplementary Figure 1), suggesting that depletion of CTC1 by miRNA promoted telomere replication stress and telomere dysfunction, but not telomere loss.

### miR-376a-3p Promotes Replicative Telomere DNA Damage

**FIGURE 3:**
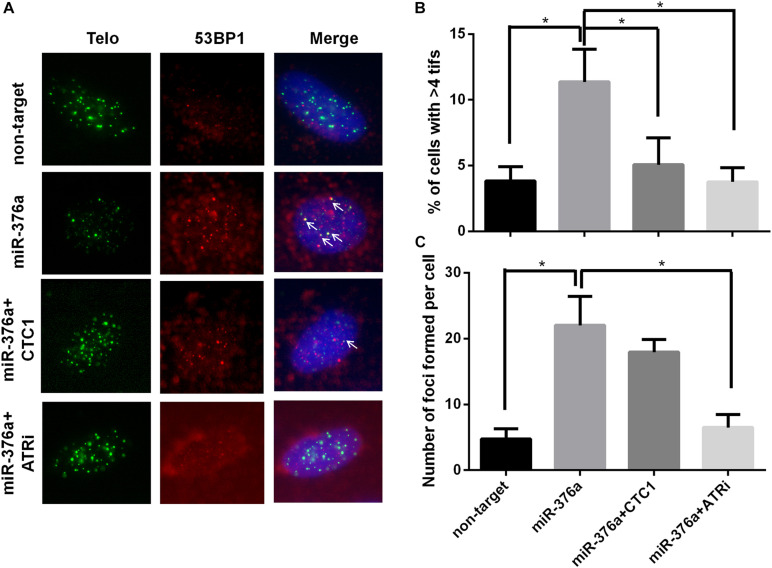
miR-376a leads to telomere damage, which relies on CTC1 expression and ataxia-telangiectasia-mutated-and-Rad3-related kinase (ATR) signal pathway activation. **(A)** Representative image showing the telomere dysfunction-induced foci (TIFs) on interphase cells with different treatments. TIFs are labeled with *white arrows*. Telomere (*green*), 53BP1 (*red*), and nuclei were stained with DAPI (*blue*). **(B)** The percentage of cells with more than four TIFs was calculated. **(C)** Graph showing the number of 53BP1 foci under different conditions. *P* values were determined by one-way ANOVA, with *P < 0.05.

Impaired telomere replication can cause telomere dysfunction, which eventually induces robust DNA damage response signals at telomeres to form telomere TIFs (Takai et al., 2003; Antushevich et al., 2014). The association of 53BP1 with the telomere signal was examined, and the result showed that the percentage of cells with more than four TIFs was increased by about threefold (from 3.8 to 11.3%) upon miR-376a-3p expression (Figure 3A). Moreover, we observed that the number of dysfunctional telomeres could be rescued by the adding back of exogenous CTC1. Ataxia-telangiectasia-mutated-and-Rad3-related kinase (ATR) signaling appears to be located in stalled replication forks to stop further fork collapse and breakage, which was mainly mediated by single-strand breaks (SSBs) (Ammazzalorso et al., 2010; Lin et al., 2018). To address the question whether the inhibition of ATR could restore the miRNA-induced telomere damage clustering, the level of telomere damage was determined with the ATR inhibitor (ATRi) treatment. To our surprise, ATR inhibition fully recovered the formation of TIFs. Combined with the SFE results, our current data indicated that miR 376a 3p could induce replication stress and SSBs rather than double-strand breaks (Figure 3B).

We have previously reported that the CST complex play a dual role in telomere DNA replication and genomic DNA replication. To investigate the effect of miR 376a 3p on general DNA replication, the number of 53BP1 foci was counted after CTC1 depletion, with or without the ATRi. Consistently, the number of 53BP1 foci was increased by sixfold with miR 376a 3p expression. However, unlike TIFs which were almost recovered by adding back CTC1, the formation of 53BP1 foci could only be partially rescued by adding back CTC1, suggesting that miR 376a 3p may also target other factors besides CTC1. Most interestingly, the overall 53BP1 foci induced by miR 376a 3p could be completely eliminated by an ATRi (Figure 3C), indicating that miR 376a 3p might have an extra target which also functions in genomic DNA replication.

### miR-376a-3p Induces Telomere Shortening Independent of Telomerase Activity

**FIGURE 4:**
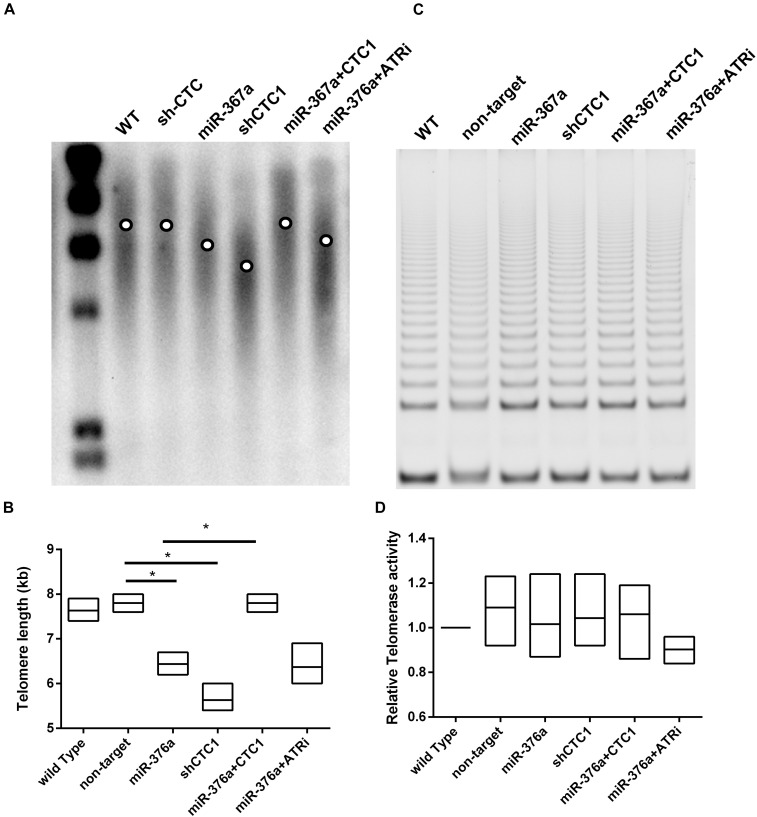
miR-376a causes telomere shortening independent of telomerase. **(A)** Telomere length was determined by TRF Southern blot; the mean telomere length in each treatment group was labeled by the *circle in the lane*. **(B)** The telomere length was calculated and graphed based on the image in graph (**A**). **(C)** Telomerase activity determined by TRAP assay is demonstrated. **(D)** The telomerase activity among different treatment groups was calculated and graphed. Statistical analysis was determined by one-way ANOVA, with *P < 0.05.

It has been reported that the depletion of CST leads to telomere signal loss and the deregulation of telomere length (Surovtseva et al., 2009). However, another study described that CST was essential in telomerase recruitment and inhibited telomere elongation (Chen et al., 2012). Given that the regulation of telomere length by the CST complex was not clear, the length of telomeres was examined here in HEK293T cells. To our surprise, the expression of miR 376a 3p led to a dramatic telomere deregulation as well as CTC1 depletion by short hairpin RNA (shRNA). The subsequent rescue experiment proved that the telomere shortening induced by miR 376a 3p could be restored by CTC1 re-expression. Interestingly, unlike the telomere dysfunction-induced foci, we observed that the ATRi treatment had no effect on telomeres in shortening recovery (Figures 4A,B). Since the recovery of telomere elongation was not dependent on the ATR signal pathway, the telomere shortening induced by miRNAs may rely on the telomere replication defects or the downregulation of telomerase. Thus, we suspect that the deregulation of telomere length may be *via* the inhibition of telomerase activity. To our surprise, there were no significant changes in telomerase activity with miR-367a-3p treatment or CTC1 depletion (Figures 4C,D).

### Genomic DNA Replication and Genome Integrity Are Also Altered by miR-367a-3p-Induced CTC1 Depletion

**FIGURE 5:**
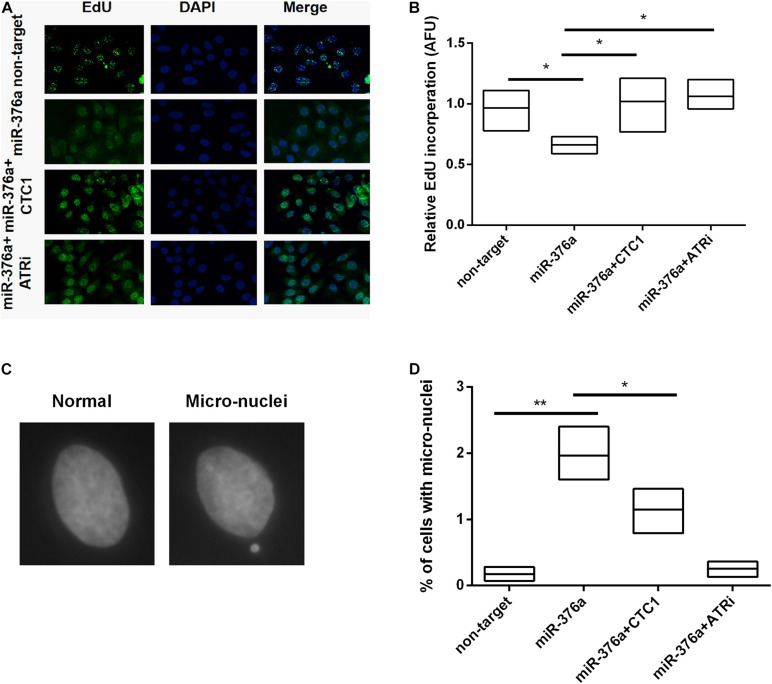
miR-376a induces genomic DNA replication deficiency. **(A)** Genomic DNA replication efficiency determined by 5-ethynyl-2′-deoxyuridine (EdU) incorporation. Incorporated EdU (*green*) and nuclei stained with DAPI (*blue*). **(B)** The relative EdU incorporation in the different treatment groups was calculated and statistically analyzed. **(C)** Representative images of normal nuclei and micronuclei. **(D)** The number of cells with micronuclei in each treatment group was calculated and the statistics of percent of cells with micronuclei was determined by one-way ANOVA. *P < 0.05 and **P < 0.01.

We have previously reported that CST was necessary for the genome-wide replication restart after fork stalling (Stewart et al., 2012). Thus, the efficiency of stalled replication fork restart was determined after hydroxyurea (HU) treatment. HU is a ribonucleotide reductase inhibitor that stalls DNA polymerization by depleting nucleotide pools (Goldstein and Kastan, 2015). After HU treatment, cells were released into the culture medium with 5-ethynyl-2′-deoxyuridine (EdU) to label the cells resuming replication. miR 376a 3p-induced lack of restarting of the stalled replication forks was verified by the decrease of EdU uptake (Figures 5A,B). As anticipated, the recovery of the fork restart was observed with CTC1 re-expression and upon ATRi treatment. To determine whether the defects in genomic DNA replication led to genome instability, hallmarks such as micronuclei were detected (Figures 5C,D). We observed that the depletion of CTC1 by either miRNA or shRNA caused a significant increase in the frequency of micronuclei formation. And the increase was barely prevented by the expression of CTC1, but largely by the treatment of ATRi (Figures 5C,D). Taken together, our findings suggested that the miR-376a-3p-induced genomic DNA replication deficiency and genome instability were *via* the depletion of CTC1 and other related factors, which relied on the activation of ATR.

### miR-376a-3p Upregulation in Rectum Adenocarcinoma Leads to the Decrease of CTC1and Poor Survival Outcome

**FIGURE 6:**
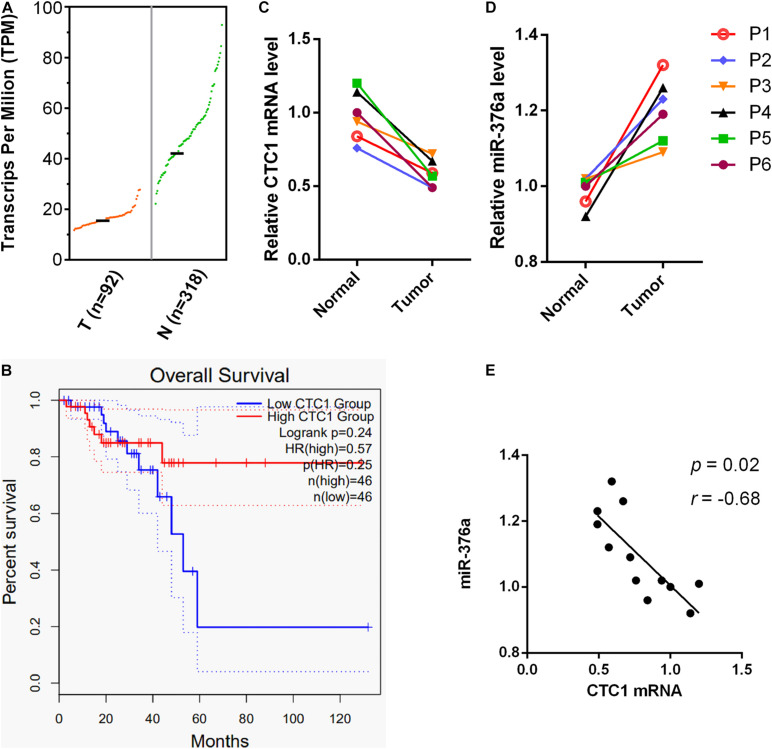
miR-376a expression is correlated with CTC1 deregulation and poor outcomes in rectum adenocarcinoma patients. **(A)** The CTC1 messenger RNA (mRNA) transcription in rectum adenocarcinoma tumor and normal tissues was analyzed. The mRNA transcriptional data was obtained from the GEPIA2 database, with 92 tumor and 318 normal samples included. **(B)** The overall survival rates of rectum adenocarcinoma patients with high or low levels of CTC1 were demonstrated based on the 92 tumor patients from the GEPIA2 database. **(C,D)** The mRNA samples of tumor tissue and adjacent tissues from six rectum adenocarcinoma patients were obtained. The transcriptional levels of CTC1 and miR-376a were determined by quantitative real-time PCR. **(E)** The inverse relation between CTC1 and miR-376a was also observed based on the transcriptional levels of the 12 samples from the tumor and adjacent tissues of the six rectum adenocarcinoma patients tested in our current study.

To further investigate the clinical significance of miR-376a-3p in cancers, the expression of its target CTC1 in different cancers was examined in The Cancer Genome Atlas (TCGA) data portal. We observed that CTC1 was significantly downregulated in several cancer types, including rectum adenocarcinoma and uterine serous carcinoma (Figure 6A and Supplementary Figure 2). RNA sequencing (RNA-seq) datasets from 410 rectum adenocarcinoma patients from READ were obtained from TCGA. The survival analysis showed that a low CTC1 expression was associated with poor survival outcome (Figure 6B). RNA was extracted from the tumor and adjacent tissues from six patients with rectum adenocarcinoma. The results showed that the transcription of CTC1 was inhibited by the overexpression miR-376a-3p (Figures 6C,D), and their expressions were closely associated (*P* = 0.02; Figure 6E).

## Discussion

Here, we employed a bioinformatics screening approach (Supplementary Figure 3) to identify miRNAs that could target the human telomere binding protein CTC1, which is one component of the CST complex. Although 11 miRNAs were predicted by the bioinformatics tool, five of them can significantly decrease the relative luciferase activity. Two miRNAs (miR-367a-3p and miR29a-3p) that showed the best depletion efficiency were chosen for our subsequent study. The expressions of these two miRNAs led to telomere replication defects and increased telomere dysfunction-induced foci in several cell lines, exhibiting a CTC1-dependent model which may rely on the ATR signal pathway. In a further study, we identified that miR-367a-3p expression could deregulate telomere length, which could be rescued by CTC1 re-expression, but not by the ATRi treatment. Moreover, based on the database analysis and the patient study, we observed that miR-376a-3p may play essential roles in promoting rectum adenocarcinoma progression *via* downregulating CTC1. Taken together, we identified novel miRNAs which could target CTC1 to promote rectum adenocarcinoma, and our current study provided a detailed mechanism by which telomere function is regulated.

Several other miRNAs targeting telomere binding proteins, such as TRF2 and POT1, have been identified in previous studies (Luo et al., 2015; Li et al., 2020). The expressions of these miRNAs demonstrated an induced telomere dysfunction and cellular senescence *via* downregulating the target protein. The CST complex is structurally similar to replication protein A (RPA), the most abundant single-strand DNA (ssDNA) binding protein that is essential for DNA replication and repair (Niederberger, 2013; Bhattacharjee et al., 2016; Bhat and Cortez, 2018; Pokhrel et al., 2019). The depletion of CST led to telomere replication stress, including increased fragility and gradual telomere shortening (Wang et al., 2012; Zhang M. et al., 2019). Here, the telomere replication deficiency was examined. As anticipated, single-strand telomeric DNA damage induced by miR-376a-3p could be fully repressed by either ATRi treatment or CTC1 re-expression. However, the genomic DNA damage could only be restored by an ATRi, but barely by a CTC1-containing vector, suggesting that miR-376a-3p has additional targets besides its CTC1 function in genomic DNA replication or single-strand DNA damage response. miR-376a has been reported to accumulate in glioma cells and to play an essential role in glioblastoma (GBM) cell invasion and migration. This effect was regulated by its direct targeting to RAP2A and its concomitant inability to target the autocrine motility factor receptor (AMFR) (Choudhury et al., 2012). Moreover, tumor genes including c-Myc, KLF15, and NRP1 were reported to be targeted by miR-376a, which may explain why the re-expression of CTC1 cannot fully rescue the miR-376a overexpression-induced defects (Yang et al., 2017; Wang et al., 2018; Zhang L. et al., 2019). The subsequent EdU incorporation and micronuclei formation study further established that miR-376a-3p has an extra target which is in charge of genomic DNA replication, and it is consistent with the multiple targeting properties of miRNAs. In contrast, the telomere length shortening induced by miR-367a-3p could only be rescued by CTC1 overexpression, but not ATRi treatment. Telomere shortening may be induced by telomere replication defects, direct telomere loss, or downregulation of telomerase activity in cancer cells. Since no significant telomere loss or reduced telomerase activity was observed, we suspected that the shortening may have resulted from the replication dysfunction of the telomere. There is an additional telomere maintenance mechanism, named alternative lengthening of telomeres (ALT), that requires the participation of ATR and its partners (Flynn et al., 2015; Sobinoff and Pickett, 2017). Given that the phenomenon of miRNAs inducing telomere shortening was not a response for the ATRi treatment, we suspected that the observed telomere dysfunction was not due to the inhibition of ALT.

Germline CTC1 mutation has also been observed in DC and acquired bone marrow failure patients (Shen et al., 2019). DC is a rare inherited bone marrow failure disorder caused by aberrant telomere shortening. It has been reviewed that the incidence of cancer in DC patients with abnormal telomere is dramatically high compared to the control group, including head and neck squamous cell carcinoma (HNSCC), skin SCC, anogenital cancer, stomach cancer, esophagus cancer, and lymphomas, as well as acute myeloid leukemia (AML) (Alter et al., 2009). TCGA expression analysis reveals that CTC1 mutation or CTC1 downregulation is highly associated with adrenocortical carcinoma, kidney chromophobe, rectum adenocarcinoma, uterine carcinosarcoma, and some other types of cancer formation. Additionally, miR-376a-3p has been reported to be positively related with colorectal cancer and endometrial cancer progression (Shen et al., 2020; Wang et al., 2020). Here, we observed that CTC1 expression was negatively associated with miR-376a-3p in adrenocortical carcinoma patients and that the overall survival is dramatically decreased with low CTC1 levels, suggesting that miR-376a-3p may stimulate adrenocortical carcinoma by targeting CTC1. Consequently, CTC1 regulation by miR-376a-3p may provide adaptive mechanisms for understanding the tumor progression. Together, our findings underscore the importance of miRNAs in controlling cell senescence or tumor generation *via* CST-mediated telomere replication. Moreover, the miR-376a in serum may be considered a hallmark of specific types of cancers and may act as a potential target in cancer therapy.

## Materials and Methods

### Gene Expression Analysis in Tumor

Gene expression profiles and the corresponding clinical information were collected from TCGA by applying the “general expression analysis” module of GEPIA2. Total mRNA samples of rectum adenocarcinoma and adjacent tissues were obtained from Telocom Company (Tianjin, China). The relative mRNA expressional levels of CTC1 from six rectum adenocarcinoma and six adjacent tissues were evaluated by quantitative real-time PCR.

### Cell Lines and Compounds

The human cervix epithelioid cell line HeLa1.2.11 was obtained from Carolyn Price Lab (Cincinnati, OH, United States) and cultured in RPMI 1640 medium. HEK293T and colon cancer cell line HCT116 cells were purchased from Tianjin Heshui Biological Industries and maintained with Dulbecco’s modified Eagle’s medium (DMEM). All of the cells were grown with 10% fetal bovine serum and 1% penicillin–streptomycin in a humidified incubator at 37°C and 5% CO_2_. The fresh medium was changed every 2 days. Cells were treated with the ATRi MKU55933 (Selleck Chemical) at a final concentration of 10 μM.

### Antibodies

The antibodies used for immunofluorescence were as follows: the 53BP1 rabbit antibody (NB100-304) was purchased from Novus Biologicals and used at a ratio of 1:5,000, while the goat anti-rabbit secondary antibodies with Alexa Fluor 555 were purchased from Invitrogen and used at a dilution of 1:2,000.

### Vectors

To express human miRNA, the vectors used in this experiment were constructed according to a method described previously (Gu et al., 2019). Specifically, the human CTC1 3′-UTR was amplified by PCR and, subsequently, the amplified fragments were inserted into the downstream of the Renilla luciferase reporter in the psiCHECK-2 vector (Promega, Madison, WI, United States). Quick-change PCR was conducted to mutate the seed region of miR-367a in the CTC1 3′-UTR. The pLKO.1 construct TRCN0000129086-D7 (5′-GATCAGAAGGTTCACCTCATT) containing shRNA targeting human CTC1 (C17ORF68, NM_025099) was purchased from Open Biosystems.

### Dual-Luciferase Assay

To identify the effect of candidate miRNAs targeting CTC1, the dual-luciferase vector and the miRNAs were co-transfected into HEK293T cells by using Lipofectamine 2000. The dual-luciferase reporter assay was performed according to the manufacturer’s instructions (Promega, E1960). Briefly, the HEK293T cells were inoculated into a 24-well plate 24 h prior to the infection. Then, the CTC1 3′-UTR (150 ng) was transfected into the HEK293T cells with the specific individual miRNA expression vector (450 ng) or the negative control vector, respectively. After 48 h, cells were harvested and lysed to detect the firefly and Renilla luciferase activities using the Dual-Luciferase Reporter Assay Kit (Promega). Then, the ratio of Renilla luciferase activity to FL activity was calculated. Finally, luciferase activity was obtained by normalizing the ratio of cells transfected with an individual miRNA expression vector to cells transfected with the miRNA negative control vector.

### Immunofluorescence and FISH

Cells were inoculated into chamber slides. After corresponding treatment, they were taken out and fixed at room temperature with 4% paraformaldehyde for 15 min, washed three times with phosphate-buffered saline (PBS) for 5 min each time, and were treated with 0.15% Triton X-100 for 15 min to permeate. After washing with PBS, they were sealed with 10% bovine serum albumin (BSA) at 37°C for 1 h and incubated with primary antibodies at 4°C overnight. The humidified chamber was taken out the next day and washed with PBS three times, then incubated with secondary antibody at room temperature for 1 h. After washing with PBS, they were stained with DAPI (Vector Labs) for visualization. Images were taken under a Nikon ECLIPSE Ti fluorescence microscope with a × 100 objective.

The FISH experiment used in this article was in accordance with a method previously described (Vinchure et al., 2020), to detect the telomeres of metaphase chromosomes in the cells fixed with methanol/acetic acid. The detecting probes were as follows: fluorescein isothiocyanate (FITC) G-strand probe (5′-CCCTAACCCTAACCCTAA, Biosynthesis) or TelG-Cy3PNA C-strand probe (5′-GGGTTAGGGTTAGGGTTA, Biosynthesis). Images were taken at a constant exposure time and quantified by counting the MTSs and SFEs with the eyes.

### Quantitative Real-Time PCR

Total RNA from the cell samples was extracted and purified using the RNeasy kit (Qiagen). The efficiency of CTC1 knockdown was detected using the one-step RT-qPCR HotStart-It kit (USB) following the manufacturer’s instructions. The primers used for quantitative real-time PCR (qRT-PCR) were: CTC1F4, 5′-TCTACCCAGAGAGTGCTTCCTGC; CTC1R4, 5′-GGACCTGCACGATGATGGACAC.

## Data Availability Statement

The raw data supporting the conclusions of this article will be made available by the authors, without undue reservation.

## Author Contributions

YL, QL, FW, and QW designed the work. YL, XZ, and BW performed the experiments. MZ, JW, YW, CX, and LD contributed to the data analysis and manuscript preparation. YL and XZ prepared the manuscript. All authors contributed to the article and approved the submitted version.

## Conflict of Interest

The authors declare that the research was conducted in the absence of any commercial or financial relationships that could be construed as a potential conflict of interest.
