# Supplementary figures and images for: miR-376a Provokes Rectum Adenocarcinoma Via CTC1 Depletion-Induced Telomere Dysfunction
Source: Front Cell Dev Biol. 2021 Apr 16;9:649328. doi: 10.3389/fcell.2021.649328 (PMC8085492; doi:10.3389/fcell.2021.649328)

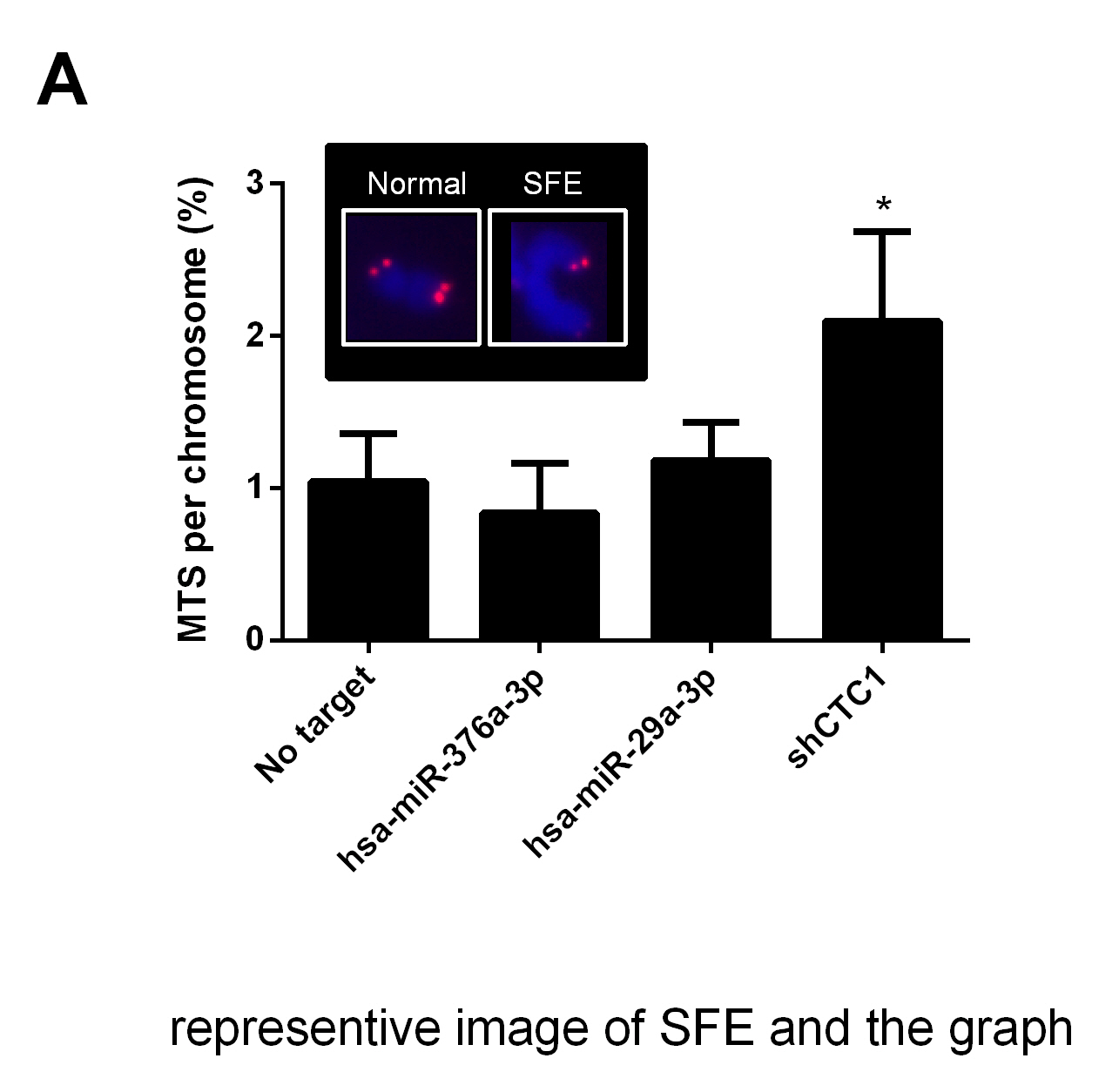

Supplement: Supplementary file 1 [file Image_1.JPEG]

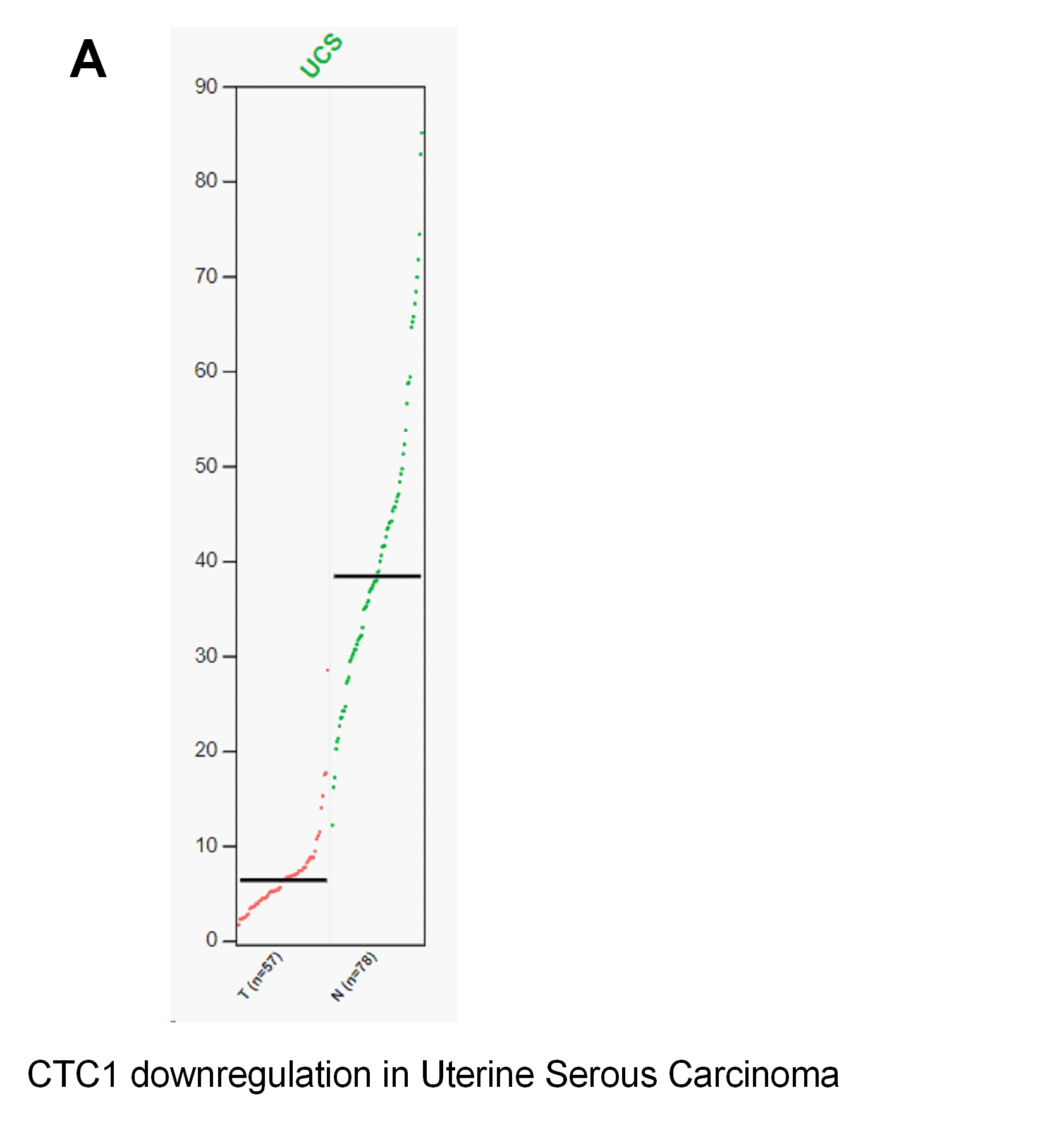

Supplement: Supplementary file 2 [file Image_2.JPEG]

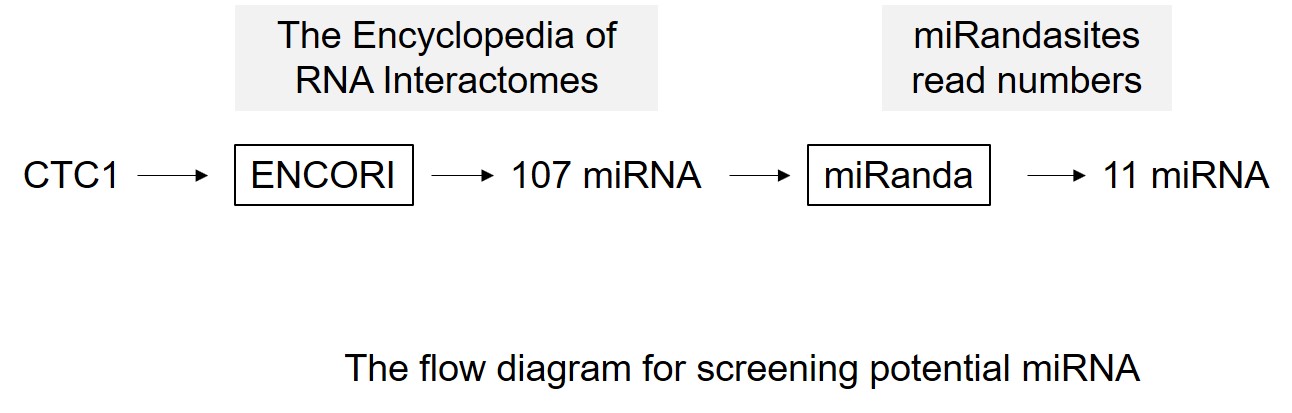

Supplement: Supplementary file 3 [file Image_3.JPEG]
